# Grafted Chitosan-Hyaluronic Acid (CS-g-poly (MA-co-AN) HA) Complex Inhibits Fluconazole-Resistant *Candida albicans* Biofilm Formation

**DOI:** 10.3390/antibiotics11070950

**Published:** 2022-07-15

**Authors:** Chaitany Jayprakash Raorane, Divya Shastri, Asrafali Shakila Parveen, Rajesh Haldhar, Vinit Raj, Seong-Cheol Kim

**Affiliations:** 1 School of Chemical Engineering, Yeungnam University, Gyeongsan 38541, Korea; chaitanyaraorane22@gmail.com (C.J.R.); shakilaasraf@gmail.com (A.S.P.); rajeshhaldhar.lpu@gmail.com (R.H.); 2 School of Pharmacy, Yeungnam University, Gyeongsan 38541, Korea; divyashastri8@gmail.com

**Keywords:** chitosan, hyaluronic acid, grafting, complex, antibiofilm

## Abstract

Fungal resistance that leads to the failure of drug therapy due to biofilm development is a major clinical challenge. Various polysaccharides have been used to control biofilm formation by drug-resistant fungi, and this study was undertaken to develop chitosan (CS)-modified materials and evaluate their abilities to inhibit *Candida* biofilm growth. CS was grafted with methacrylamide (MA) and acrylonitrile (AN) and, to improve its application characteristics further, was grafted with hyaluronic acid to produce CS-g-poly (MA-co-AN) HA complex. Grafting and complex formation were confirmed using spectroscopic techniques. CS-g-poly (MA-co-AN) HA was tested to investigate its ability to inhibit *Candida albicans* biofilm formation and showed significant antibiofilm activity at 200 µg/mL. Additionally, CS-g-poly (MA-co-AN) HA did not have any toxic effect on *Caenorhabditis elegans*. Thus, this study provides an innovative means of preventing microorganism-associated biofilm formation.

## 1. Introduction

The development and spread of fungal resistance to well-known antifungal drugs are increasing concerns about the possibility of a global infection crisis [1]. In past decades, natural compounds have provided leads for antifungal discovery [2,3]. However, synthetic structural modifications of natural compounds allow the production of nearly limitless numbers of related compounds with antifungal properties [4]. Nevertheless, due to the widespread usage of antifungal agents, several fungal strains have developed resistance [5,6]. As a result, various approaches have been devised to improve drug effectiveness and overcome resistance-related issues. In particular, polysaccharide hydrogels and nanocarriers have been used as drug carriers because of their remarkable abilities to prevent biofilm formation [7,8].

Chitosan (CS) is a natural polymer that is produced by the deacetylation of chitin and is abundant in nature. Also, chitosan has been widely used as a drug delivery material, an adsorbent, and for food packaging because of its non-toxic, low cost, degradable, and compatible nature [5,9,10,11]. On the other hand, hyaluronic acid (HA) is another biocompatible polysaccharide that is widely employed as a drug delivery material [12,13]. Furthermore, chitosan and HA have been modified to improve drug solubilities and their drug entrapment efficiencies. Several monomers are used to modify the functional groups of these two polysaccharides. Acrylonitrile is the monomer that is most commonly used to facilitate the grafting of polysaccharides and the synthesis of hydrogels [14]. Acrylonitrile is used to produce plastics, and chitosan that is grafted with acrylonitrile (AN) has promising antibacterial activities. Furthermore, an AN-modified cellulose has been reported to adsorb chromium VI ions from the solution. Methyl acrylamide (MA) is another biocompatible monomer which has been reported to enhance hydrogel absorption [9,15]. Furthermore, these monomer-based modifications of CS and HA are degradable and have antibacterial properties, and thus, low environmental impacts.

**Scheme 1:**
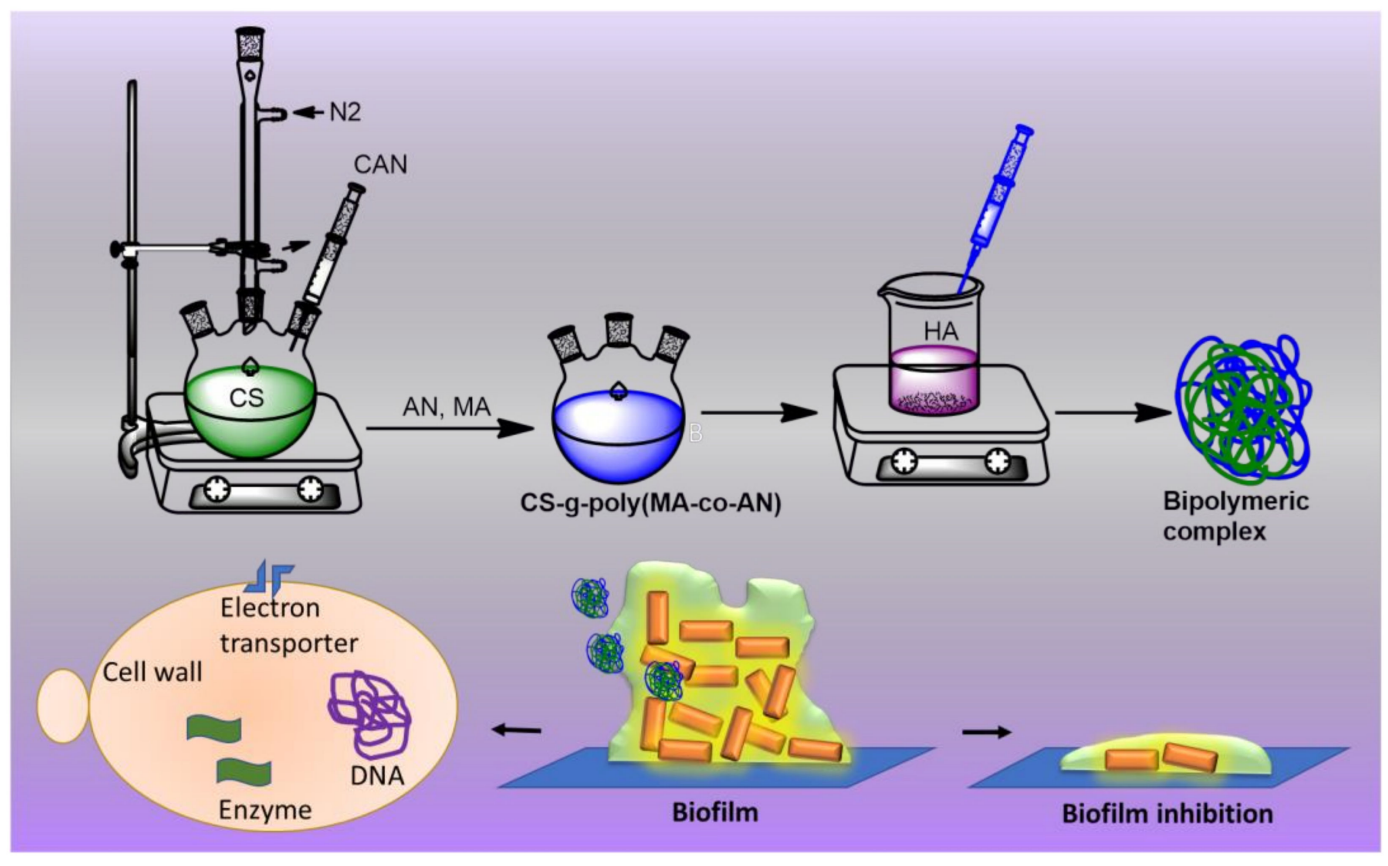
Schematic of the strategy that was used to modify CS and investigate its effects on *C. albicans* biofilms. In which CS used a biopolymeric matrix for binary grafting with AN, and MA to improve its physicochemical properties of it using the grafting redox method. Later, the grafted CS solution was added into an aqueous solution of HA to form the ionic complex matrix because HA possesses the negative charge while modified CS showed the positive charge. Finally, the biopolymeric matrix was employed for antibiofilm activity against the *C. albicans* strain.

Inspired by the significant uses of CS and HA and their complexes, we have hypothesized that the complex of modified CS with HA might work as an alternative biopolymeric material for antifungal drug delivery as a polymeric matrix with improved antibiofilm potency. Hence, we considered that grafted CS-HA complexes might penetrate biofilms and improve the antibiofilm activity of CS and possibly provide a means of treating multidrug-resistant biofilm-associated infections (Scheme 1). Thus, the main objective of this study was to graft CS with MA and AN and to later incorporate hyaluronic acid to form a CS-g-poly (MA-co-AN) HA) complex and investigate its antibiofilm potency. 

**Scheme 2:**
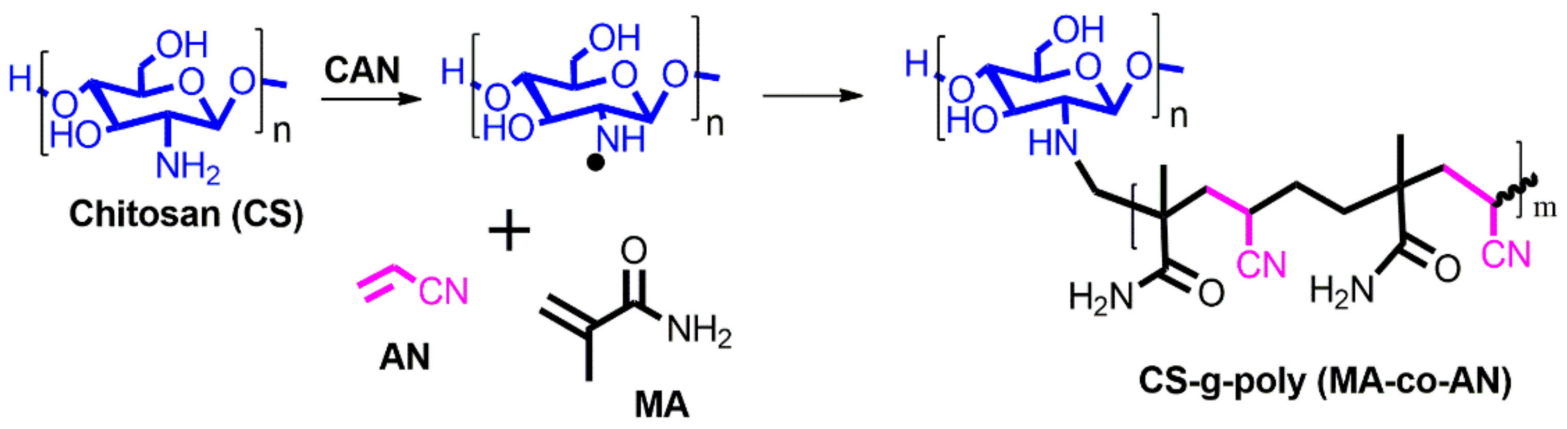
Synthesis of binary grafting of CS-g-poly (MA-co-AN) by a free radical redox reaction, whereas a chemical initiator ceric ammonium nitrate (CAN) was used along with chitosan (CS) and two monomers, namely methacrylamide (MA) and acrylonitrile (AN).

**Scheme 3:**
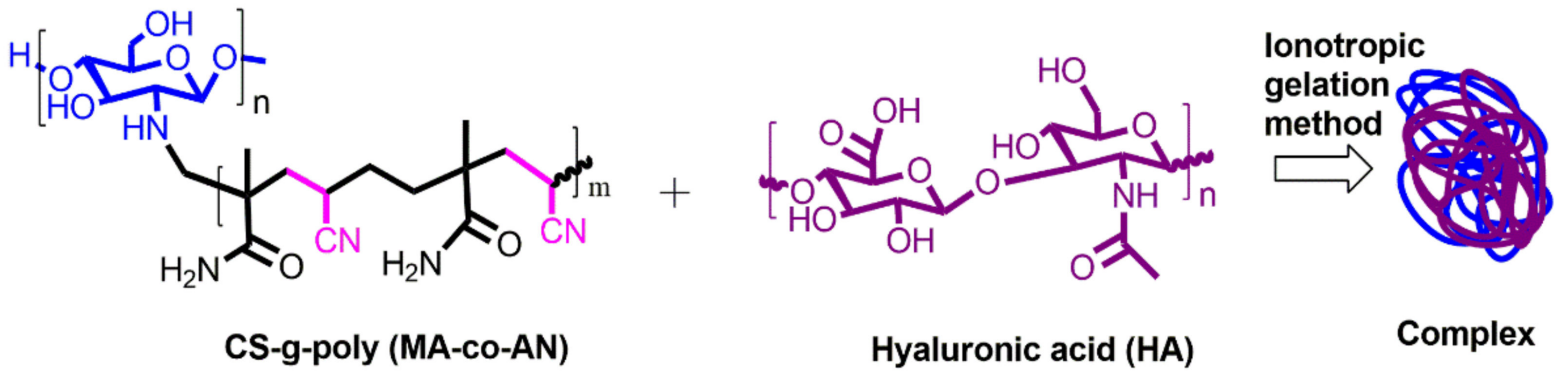
Preparation of CS-g-poly (MA-co-AN) HA complex using an ionotropic gelation method. The binary grafted CS-g-poly (MA-co-AN) was dissolved into a 1% acetic acid solution and the aqueous solution of HA was prepared in distilled water. The binary grafted CS solution was added dropwise into the HA acid solution to form the ionic complex.

To achieve the main objective, CS was first modified with AN and MA. HA was then embedded into this modified CS to generate a CS-g-poly (MA-co-AN) HA complex using the ionotropic gelation method, which is an eco-friendly and relatively cheap method (Scheme 2 and Scheme 3). The grafting of CS and ionic complexation between CS-g-poly (MA-co-AN) and HA was confirmed by proton nuclear magnetic resonance (^1^H NMR) and Fourier-transform infrared (FTIR) spectroscopy. The surface architectures and morphologies of the grafted CS and CS-g-poly (MA-co-AN) HA were investigated by scanning electron microscopy (SEM). In addition, thermogravimetric (TGA) differential scanning calorimetry (DSC) was used to investigate the thermal behavior of the grafted CS. To investigate the antibiofilm potency of CS-g-poly (MA-co-AN) HA complex in vitro, we used a *C. albicans* biofilm assay. SEM and light microscopy were employed to examine the effect of CS-g-poly (MA-co-AN) HA on hyphae formation by *C. albicans*. As result, CS-g-poly (MA-co-AN) HA complex could have adhered on the *C. albicans* surface, which is probable to enter the membrane owing to improved permeability. Hence, CS-g-poly (MA-co-AN) HA complex is expected to exhibit outstanding efficacy with reduced drug resistance in the treatment of biofilm-associated *C. albicans* infections, and also the prepared complex can be used as an alternative biopolymeric matrix to the antifungal drug delivery. 

## 2. Results and Discussion

### 2.1. Characterization of the CS-g-poly (MA-co-AN)-HA Complex

#### 2.1.1. Zeta Potentials

**Figure 1:**
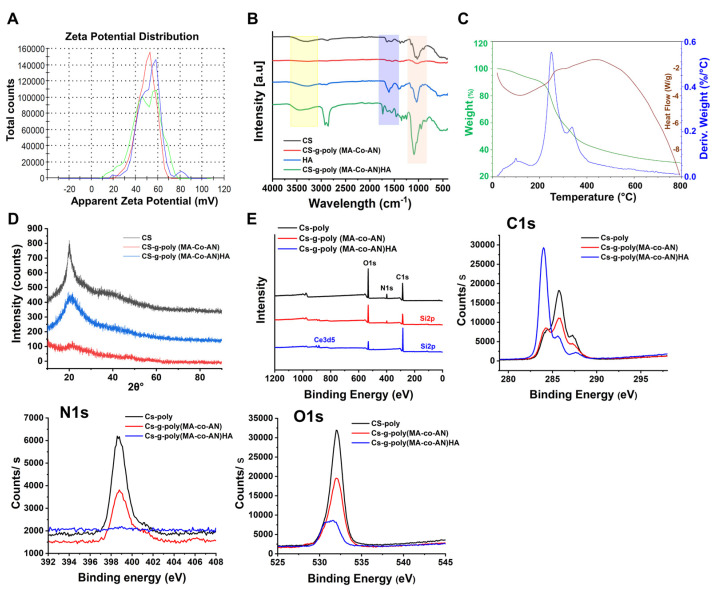
Characterization of the CS-g-poly and CS-g-poly(MA-co-AN) HA; (**A**) zeta potential (mV) of CS-g-poly (MA-Co-AN), (**B**) FTIR analysis (black, red, blue, and green colors show the CS, CS-g-poly (MA-co-AN) HA, and CS-g-poly(MA-co-AN) HA, respectively), (**C**) simultaneous DSC−TGA of CS-g-poly (MA-Co-AN) HA, (**D**) XRD analysis of CS-g-poly, CS-g-poly (MA-Co-AN), and CS-g-poly (MA-Co-AN)-HA (black, red, and blue show the CS, CS-g-poly (MA-Co-AN), and CS-g-poly(MA-Co-AN) HA, respectively), (**E**) XPS analysis of CS, CS-g-poly (MA-Co-AN), and CS-g-poly (MA-Co-AN)-HA, and their C1s, N1s, and O1s binding energy spectra, (black, red, and blue show the CS, CS-g-poly (MA-Co-AN), and CS-g-poly(MA-co-AN) HA, respectively).

The zeta potentials of CS, CS-g-poly (MA-co-AN), and CS-g-poly (MA-Co-AN) HA were measured using the zeta sizer Nano ZS (Malvern Instruments, Shanghai, China) (Figure 1A). The zeta potentials of CS, CS-g-poly (MA-co-AN), and CS-g-poly (MA-co-AN) HA were +45.4 ± 4.23, +49.5 ± 8.70, and +34.7 ± 5.44 mV, respectively. The zeta potentials of polymeric materials play significant roles in interactions with anions such as HA, and these interactions underpin the ionotropic gelation method that was used to produce CS-g-poly (MA-co-AN)-HA. CS has a positive surface charge. After grafting MA and AN onto the NH_2_ groups of CS, the binary chain formed on the backbone of modified CS. These monomers on the backbone of CS developed a high positive charge after the grafting compared to CS. It has been reported that unmodified CS and HA acid nanocarriers in the presence of pentasodium tripolyphosphate showed zeta potential −1.1 to −40.5 mV [16]. In the present study, the complex showed an improved positive zeta potential, suggesting that this method is suitable to develop positive charge-based biomaterials. 

#### 2.1.2. FTIR and XRD Analysis of CS-g-poly (MA-co-AN) HA Complex

Figure 1B shows the FTIR spectra of CS, HA, CS-g-poly (MA-Co-AN), and CS-g-poly (MA-Co-AN) HA. For CS, OH stretch was observed at 3285.68 cm^−1^. This peak was broadened due to overlap with the NH peak at 3357 cm^−1^. The peak at 2870.4 cm^−1^ was attributed to C-H asymmetric stretch and the peak at 1152.6 cm^−1^ to an asymmetric stretch of C-O-C bridges. C-O stretch was observed at 1028.30 cm^−1^, and N-H bending of primary amines at 1645.7 cm^−1^. Extensional vibration of the CH band was observed at 1555.4 cm^−1^. CS-g-poly (MA-Co-AN) showed a broad stretch peak at 3292.6 cm^−1^ of low intensity because the NH groups of the monomers overlapped with the hydroxyl groups of CS. C-H asymmetric stretch was observed at 2873.6 cm^−1^. CN and primary amine N-H bending vibrations were observed at 1247.5 cm^−1^ and 1642.4 cm^−1^, respectively. The C-O stretching band was observed at 1020.4 cm^−1^. In the FTIR spectra of HA, the broad peak at 3276.32 cm^−1^ was attributed to O-H vibration and the peak at 2896 cm^−1^ to asymmetric vibration of C-H. The sharp peak at 1609.07 cm^−1^ was attributed to the carboxylic group of HA. The FTIR spectra of CS-g-poly (MA-Co-AN) HA had a broad OH peak at 3471 cm^−1^. C-H symmetric and non-symmetric stretch were observed at 2919.8 cm^−1^ and 2857.59 cm^−1^. The higher intensity of these peaks suggested that monomers had successfully attached to CS. A sharp C=O stretch peak at 1734.57 cm^−1^ confirmed the presence of monomer substitution on the surface of CS. Additionally, two peaks at 1678 and 1550.92 cm^−1^ were observed. The CN peak was observed at 1248.18 cm^−1^ and the C-OH stretch at 1093.14 cm^−1^. These peaks confirmed that AA and MA had been grafted onto the CS backbone.

XRD analysis was used to determine whether CS, CS-g-poly (MA-co-AN), and CS-g-poly (MA-co-AN) HA were crystalline or amorphous. As shown in Figure 1D, CS showed a high-intensity peak at 20.01 2θ, but after grafting with MA-AA, this peak broadened and shifted to 21.51 2θ with slightly reduced intensity, suggesting a more amorphous nature. CS-g-poly (MA-co-AN)-HA showed a low-intensity peak at 19.80 2θ, indicating a highly amorphous nature. Grafting of the CS backbone enhanced the amorphous nature of CS-g-poly (MA-co-AN)-HA.

#### 2.1.3. DSC–TGA of CS-g-poly (MA-co-AN) HA

DSC-TGA analysis was conducted to obtain insight into the decomposition of CS-g-poly (MA-co-AN) HA. CS-g-poly (MA-co-AN) HA started to decompose at 251.96 °C due to the loss of adsorbed moisture; exothermic decomposition of the polymeric backbone started at 341.82 °C (50% weight loss) (Figure 1C) while CS showed a polymeric backbone at 329.86 °C [8]. The improved thermal stability of CS-g-poly (MA-co-AN) HA was attributed to the presence of the biopolymer. At 792.66 °C, 70% of CS-g-poly (MA-co-AN) HA decomposed, whereas this occurred at 540.18 °C for CS. Generally, CS underwent thermal decomposition in two ways, that is, by the loss of water from 25 to 150 °C and by CS decomposition at 200 to 500 °C [17]. Consequently, CS-g-poly (MA-co-AN) HA showed improved thermal stability owing to the polymeric backbone formation of AN and MA monomers. Hence, improving the thermal stability of the complex might be useful to protect it from oxidation. 

#### 2.1.4. XPS and NMR Analysis

XPS is used to measure the electronic and chemical states of elements and to determine binding energies. We analyzed C, N, and O patterns, as shown in Figure 1E. For CS, binding energies of 285.75, 398.74, and 532.06 eV represented C1s, N1s, and O1s, respectively. The XPS spectrum of CS-g-poly (MA-co-AN) exhibited corresponding peaks at 285.64, 398.82, and 532.04 eV, which showed the binding energy with a slightly increased % of C1s atoms. In addition, extra peaks were observed at ~150 and 200 eV. The N1s signal was located at 398.82 eV and deceased sharply in CS-g-poly (MA-co-AN) HA, suggesting interactions between the amine groups of CS-g-poly (MA-co-AN) and HA. High-resolution C1s XPS offers an insight into the chemical binding states. Alterations in the peak intensities and changes in atom percentages confirmed the modification of CS. A small-signal that was located at 287.9 eV corresponded to C-O or C-N while peaks at 285.75 and 284.1 eV were attributed to C-C/C-H. After grafting, the intensities of C1s peak at 285.75 and 287.9 eV deceased, indicating bond formation between the NH_2_ groups of CS and CH chain of monomer. However, the intensity of the C1s peak sharply increased after the inclusion of HA. Also, CS-g-poly (MA-co-AN) HA showed binding energy peaks of C1s, N1s, and O1s at 283.99, 398.94, and 531.29 eV, respectively, with an increased 84.43% of C1s atomic, whereas N1s and O1s atoms percentages were lower than for CS.

The high-intensity N1s and O1s peaks at 398.74 and 532.06 eV in CS decreased after grafting, indicating amine/monomer bond formation. Additionally, the N1s signal was low for CS-g-poly (MA-co-AN) HA, suggesting that the NH_2_ of CS interacted ionically with the carboxylic group of HA. The intensity of O1s peaks in CS-g-poly (MA-co-AN) was lower than in CS, and CS-g-poly (MA-co-AN) HA had a low-intensity O1s peak, indicating an ionic interaction between the HA carboxylic group and the hydroxyl and ketone groups of modified CS.

**Figure 2:**
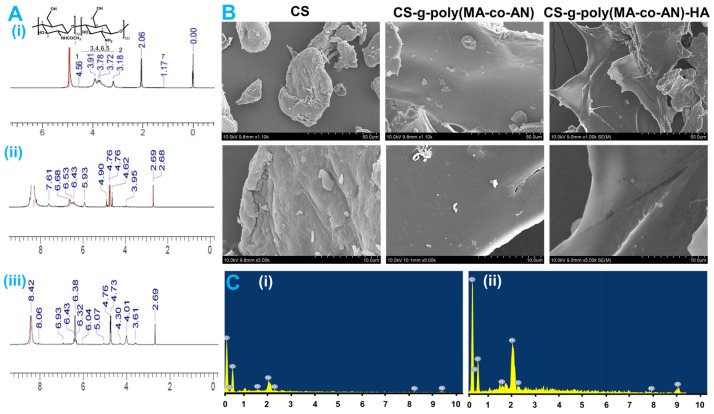
Representing the NMR analysis and surface characterization; (**A**) ^1^H NMR spectra of the (**i**) CS, (**ii**) CS-g-poly (MA-co-AN), and (**iii**) CS-g-poly (MA-co-AN) HA; (**B**) Surface characterization by SEM, (**C**) EDX mapping of (**i**) CS-g-poly (MA-co-AN), and (**ii**) CS-g-poly (MA-co-AN) HA.

^1^H-NMR (600 MHz, D_2_O: d-acetic acid) was used to confirm the grafting of AN and MA onto the backbone of CS (Figure 2A(i–iii)). ^1^H-NMR spectra of CS showed an NH_2_ proton peak at 4.56 δ ppm and aliphatic methylene proton peaks at 3.91–3.72 δ ppm. Also, a single signal was observed at 3.18 δ ppm, confirming the methylene proton of the aliphatic cyclic ring. A methyl peak was observed at 1.17 δ ppm. In the ^1^H-NMR spectra of CS-g-poly (MA-co-AN), extra peaks were observed at 7.61 δ ppm and 6.68–6.43 δ ppm, confirming the presence of the amide groups and CH protons of monomers. The extra peak at 5.93 δ ppm confirmed the bond formation of the NH_2_ group. Assigned proton peaks confirmed the grafting of AN and MA onto the CS backbone. The NMR spectrum of CS-g-poly (MA-co-AN) HA contained additional peaks at 4.01, 4.30, and 5.07 δ ppm, which confirmed the presence of CH and NH protons of HA (Figure 2A(iii)). The peak at 3.61 δ ppm confirmed the presence of CH_3_. These observations confirmed that the HA carboxylic acid group interacted ionically with CS-g-poly (MA-co-AN).

#### 2.1.5. SEM and EDX Analysis of Modified CS

SEM analyses of CS, CS-g-poly (MA-co-AN), and CS-g-poly (MA-co-AN) HA were carried out to investigate surface architecture changes that were caused by AN and MA grafting and the incorporation of HA. As shown in Figure 2B, the surface morphology of CS-g-poly (MA-co-AN) was smoother and more homogeneous than that of CS, and CS-g-poly (MA-co-AN) HA had a smoother, more homogeneous surface CS. This analysis indicated that CS-g-poly (MA-co-AN) HA formed a homogeneous structure with HA. In addition, EDX analysis of CS-g-poly (MA-co-AN) and CS-g-poly (MA-co-AN) HA revealed carbon, oxygen, and nitrogen atomic concentrations of 51.18, 37.49, and 10.33, and 52.26, 25.45, and 22.28, respectively (Figure 2C(i,ii)).

### 2.2. Antibiofilm Potency of CS-g-poly (MA-co-AN)-HA against C. albicans

**Figure 3:**
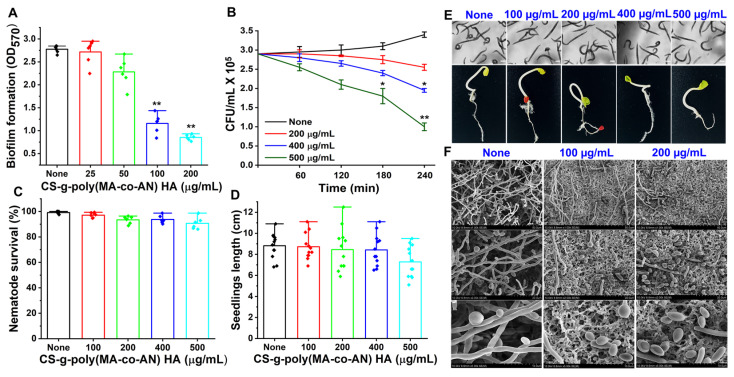
Appraisal of the antifungal and antibiofilm potency of CS-g-poly (MA-co-AN) HA complex against *C. albicans* DAY185. (**A**) Biofilm development of *C. albicans* DAY185 in the presence of CS-g-poly (MA-co-AN) HA. (**B**) Rapid killing of *C. albicans* by CS-g-poly (MA-co-AN) HA. After incubation of 24 h with CS-g-poly (MA-co-AN) HA, the cultures were plated and CFUs were counted. (**C**) Bar graph indicating the % of nematodes survivals that were treated by CS-g-poly (MA-Co-AN) HA polymer composite for seven days. (**D**) Bar graph demonstrating the effects of CS-g-poly (MA-co-AN) HA polymer composite on seedlings length after seven days. (**E**) Effects of CS-g-poly (MA-co-AN) HA polymer composite on *C. elegans* and seed germination of *R. raphanistrum*. Scale bar =100 μm. (**F**) SEM observation of *C. albicans* biofilms. The results are described as means ± SDs of triplicate. The error bars show the standard deviations. ** *p* < 0.01 and * *p* < 0.05 vs.

Biofilm assays were used to examine the antibiofilm potency of CS-g-poly (MA-co-AN) HA against *C. albicans* DAY185. Figure 3A shows dose-dependent antibiofilm inhibition after treatment with CS-g-poly (MA-co-AN) HA at 25, 50, 100, or 200 µg/mL. CS-g-poly (MA-co-AN) HA at 100 µg/mL for 24h inhibited biofilm formation by 60%. Furthermore, at 200 µg/mL, CS-g-poly (MA-co-AN) HA inhibited *C. albicans* biofilm formation by 72% without adversely affecting planktonic cell growth. The MIC of CS-g-poly (MA-co-AN) HA was found to be 350 µg/mL against *C. albicans*. The biofilm development is a survival mechanism of fungi that is more challengeable in wound infection [18,19]. Hence, the biofilm formation leads to an improved drug resistance risk or sensitivity for the marketed medications. The prepared complex might be effectively working against the *C. albicans*-associated biofilm and also can be used as a biopolymeric material for the drug delivery of antifungal drugs to improve the effectiveness and reduce the drug resistance-related issues owing to its antibiofilm potency. Thus, CS-g-poly (MA-co-AN) HA inhibited *C. albicans* biofilm but not planktonic cell growth. Hence, this complex might be used against biofilm-associated infection when it may be used with the antifungal drug because some of the antifungal drugs develop resistance in the presence of biofilms. The selective biofilm inhibition might be useful for the solution of biofilm-associated infections in the case of resistant drug molecules owing to biofilm. 

### 2.3. Rapid Killing Activity of CS-g-poly (MA-co-AN) HA

The rapid killing assay revealed CS-g-poly (MA-co-AN) HA at 400 µg/mL required 4 h to reduce cell viability by 30%. At 500 µg/mL for 3- or 4-h CS-g-poly (MA-co-AN) HA reduced *C. albicans* viability by >40% and >60%, respectively (Figure 3B). These results suggest that at higher concentrations CS-g-poly (MA-co-AN) HA could be used to control the growth of *C. albicans* DAY185.

### 2.4. SEM Analysis of CS-g-poly (MA-Co-AN) HA Treated C. albicans

An SEM showed that CS-g-poly (MA-co-AN) HA in PDB medium at 0–200 μg/mL repressed hyphal transition on nylon membranes (Figure 3F). On untreated membranes, large hyphal cells were observed, whereas CS-g-poly (MA-co-AN) HA-treated membranes had more yeast cells and only rare hyphae. Furthermore, cell-aggregation and hyphal results were in line with the detected antibiofilm activity of CS-g-poly (MA-co-AN) HA. Hence, CS-g-poly (MA-co-AN) HA prevented hyphal development, biofilm formation, and cell aggregation of *C. albicans* DAY 185 (Figure 3F). Overall, these observations show that CS-g-poly (MA-co-AN) HA effectively inhibited cell aggregation, hyphae formation, and decreased biofilm formation by *C. albicans.*

### 2.5. Safety Profile of CS-g-poly (MA-co-AN) HA as Determined by C. elegans Viability and Seed Germination Rates

In the in vivo *C. elegans* viability model, nematodes that were treated with or without CS-g-poly (MA-co-AN) HA showed similar trends (Figure 3C,E), indicating nematodes were unaffected by high doses of CS-g-poly (MA-co-AN) HA. In particular, CS-g-poly (MA-co-AN) HA at doses of <500 µg/mL did not influence survival or induce phenotypic changes in nematode morphology. To investigate the impact of CS-g-poly (MA-co-AN) HA on seed germination, *R. raphanistrum* seeds were developed on Murashige and Skoog agar containing CS-g-poly (MA-Co-AN) HA at 0–500 µg/mL. CS-g-poly (MA-co-AN) HA did not induce any phenotypic changes for the first two days, although at ≥400 µg/mL, the germination rate of *R. raphanistrum* was delayed until day seven (Figure 3D,E). Seed germination and seedling growth were marginally reduced at CS-g-poly (MA-co-AN) HA concentrations of 400 and 500 µg/mL versus the non-treated controls. In addition, the lengths of seedlings that were treated with CS-g-poly (MA-co-AN) HA at concentrations of 500 µg were significantly shorter than those of the non-treated controls (Figure 3D). CS-g-poly (MA-co-AN) HA showed similar safety profile as reported for CS-based nanocarriers and halogenated molecules in previous reports [7,8,9,10,11,12,13,14,15,16,17,18,19,20,21,22,23,24]. Thus, based on these comparative observations, it can be suggested that the prepared complex is safe for further application in drug delivery as well as environmental applications. 

## 3. Materials and Methods

### 3.1. Materials and Microbial Stains

For in vitro studies of CS-g-poly (MA-co-AN) HA complex *C. albicans* DAY185 stain (fluconazole-resistant) was generously provided by Professor J. Lee, Yeungnam University, Republic of Korea and was obtained from the Korean Culture Center of Microorganisms (http://www.kccm.or.kr/, accessed on 12 July 2022). The streaking and subculture of *C. albicans* were performed in potato dextrose agar (PDB) or potato dextrose broth (PDB). All biofilm experiments were conducted overnight at 37 °C using a fresh single colony that was inoculated into 25 mL of PDB. CS (MW~ 20–300 cps, 75%) was purchased from Sigma-Aldrich (St. Louis, MO, USA), and AN (MW 53.06, 99%) and MA (MW 85.11, 98%) from TCI (Tokyo, Japan). DMSO was supplied by Duksan Pure Chemicals (Daegu, South Korea). All the experiments were carried out using at least two individual cultures, and cell growth was measured at 600 nm.

### 3.2. Synthesis of CS-g-poly (MA-co-AN)

Grafted chitosan was produced by free radical reaction using 0.2 M methacrylamide (MA) and 0.2 M acrylonitrile (AN) (Scheme 2). Briefly, CS was dissolved in 1% acetic acid solution and 10% ceric ammonium nitrate (CAN) was added and heated for 30 min at 60 °C to initiate the redox reaction. The reaction mixture was kept at room temperature overnight, and acetone and ethanol were then added to remove unreacted material and precipitate CS-g-poly (MA-co-AN). The precipitate was then washed three times with acetone and dried at 60 °C. The final yield of CS-g-poly (MA-co-AN) that was obtained was 87.1%.

### 3.3. Fabrication of CS-g-poly (MA-co-AN) HA Complex

Synthesized CS-g-poly (MA-co-AN) (0.5%) was dissolved in a 1% acetic acid solution and a 0.5% solution of HA was prepared in distilled water. The inotropic gelation method was adopted with minor modification [7,8,9,10,11,12,13,14,15,16,17,18,19,20]. Briefly, the solution containing positively charged CS-g-poly (MA-co-AN) was added to a solution containing negatively charged hyaluronic acid (Scheme 3). The mixture was stirred continually for 4 h and then lyophilized to obtain CS-g-poly (MA-co-AN) HA as a dry powder.

### 3.4. Characterization of CS-g-poly (MA-co-AN) HA

#### 3.4.1. Zeta Potential Measurements

Synthesized CS-g-poly (MA-co-AN) HA was first dispersed in water, and the resulting dispersions were assessed for particle size, zeta potential, and polydispersity index (PDI) using a zeta sizer Nano ZS (Malvern Instruments, China). All analyses were carried out in triplicate.

#### 3.4.2. FTIR Spectroscopy CS-g-poly (MA-co-AN) HA

FTIR spectroscopy (PerkinElmer) was carried out to assess interactions between functional groups in CS-g-poly (MA-co-AN)-HA using 8 scans over the wavelength range 4000–400 cm^−1^.

#### 3.4.3. X-ray Diffraction (XRD) Analysis of CS-g-poly (MA-co-AN) HA

XRD was used to determine the amorphous or crystalline natures of CS, grafted CS, and CS-g-poly (MA-Co-AN) HA using a copper anode.

#### 3.4.4. Simultaneous DSC–TGA Analysis of CS-g-poly (MA-co-AN) HA

The thermal behavior of CS-g-poly (MA-co-AN) HA was examined by simultaneous DSC–TGA (TA Instruments; USA (TG–DTA SDT Q600)). The CS-g-poly (MA-co-AN) HA (~7 g) was heated from 20 ± 5 °C to 800 °C at a rate of 10 °C/min in a nitrogen atmosphere.

#### 3.4.5. X-ray Photoelectron Spectroscopy (XPS)

XPS analysis was performed using a K-Alpha spectrometer (Thermo Scientific, Santa Clara, CA, USA) to examine interactions between the functional groups in CS, grafted CS, and CS-g-poly (MA-co-AN) HA, and to determine O, N, and C contents using 20 Al K Alpha scans.

#### 3.4.6. ^1^H-NMR Analysis of CS-g-poly (MA-co-AN) HA

The ^1^H-NMR spectra of CS, grafted CS, and CS-g-poly (MA-co-AN) HA were obtained at 600 MHz using a VNS-600 FT-NMR, in D_2_O (99.5%). Tetramethylsilane was used as the internal standard, and chemical shifts (δ) are denoted in ppm.

#### 3.4.7. SEM and Energy-Dispersive X-ray Spectroscopy (EDX)

The surface architectures of CS, CS-g-poly (MA-co-AN), and CS-g-poly (MA-co-AN) HA were investigated by SEM (FE-SEM/EDSC (3) S-4800 Hitachi Ltd., Tokyo, Japan) using secondary electron detectors and an operating voltage of 10 kV. The samples were coated with gold for 120 s before analysis. The elemental distributions of CS-g-poly (MA-Co-AN) and CS-g-poly (MA-Co-AN) HA were analyzed by EDX.

### 3.5. Antibiofilm Potency of CS-g-poly (MA-co-AN) HA against C. albicans

Biofilm assays were performed using the crystal violet staining method in 96-well microtiter plates, as previously described [21,22], and cultures of *C. albicans* DAY185 in PDB after culture overnight at 37 °C with shaking. The cultures were re-cultured in PDB at a 1:50 dilution ratio with or without CS-g-poly (MA-co-AN) HA (0, 25, 50, 100, or 200 μg/mL) in microtiter plates at 37 °C without shaking for over 24 h, and biofilm formation was confirmed by staining with 0.1% crystal violet for 20 min. The films were then thoroughly washed with sterile distilled water and then 95% ethanol was added to each well. Absorbances were recorded at 570 nm. Assays were conducted using two independent cultures in triplicate.

### 3.6. Time-Kill Assay

To determine the killing efficacy of CS-g-poly (MA-co-AN) HA, a time-kill kinetic assay was used, as previously described [23,24]. Briefly, overnight cultures of *C. albicans* DAY 185 at a dilution of 1:50 inoculated in PDB were used for assays. The cultures were treated with (0–500 μg/mL) of CS-g-poly (MA-co-AN) HA and incubated for 4 h at 37 °C with shaking (240 rpm). At specified times, aliquots of treated cells were collected, diluted as required, plated onto PDA plates, and incubated overnight at 37 °C. Colony-forming units (CFUs) were then counted. The experiments were performed using two independent cultures in triplicate.

### 3.7. Seed Germination Toxicity Assay

The impact of CS-g-poly (MA-co-AN) HA on seed germination was examined on Murashige and Skoog agar plates as previously described [7,25] using overnight soaked seeds of *Raphanus raphanistrum*. Before the experiment, the seeds were sterilized using 1 mL of 100% ethanol and subsequent immersion in 3% sodium hypochlorite solution for 15 min. The seeds were then placed on agar plates (0.86 g/L Murashige and Skoog medium) containing 0.7% bacto-agar and CS-g-poly (MA-co-AN) HA at 0–500 μg/mL. Finally, the plates were incubated at room temperature for 7 days and photographed.

### 3.8. In Vivo Toxicity Assessment of CS-g-poly (MA-co-AN) HA Complex against C. elegans

The toxicity of CS-g-poly (MA-co-AN) HA complex was analyzed using synchronized adult *C. elegans* [*fer-15(b26);fem-1(hc17)*] nematodes as previously described [26]. Briefly, 30–40 non-infected worms were added to the wells of a 96-well dish containing M9 buffer. The wells were then treated with CS-g-poly (MA-co-AN) HA at 0–500 μg/mL and incubated for seven days at 25 °C with gentle shaking. The results are expressed as percentage nematode survivals after incubation. Viabilities were assessed by exposing the worms to LED or UV LED light [27] for 10–30 s using Optical Imaging Equipment (Nikon Eclipse 50i Republic of Korea). There were three independent experiments that were performed at each concentration.

### 3.9. The Microscopic Architecture of C. albicans Biofilms

Biofilm architecture and phenotypic changes of *C. albicans* DAY185 were observed on nylon membranes as previously described [28]. Briefly, a sterile nylon membrane (0.45 μm) 0.5 × 0.5 cm was added to 96-well plates containing *C. albicans* that were grown in PDB and treated with CS-g-poly (MA-co-AN) HA (0–200 μg/mL) for 24 h at 37 °C. *C. albicans* cells that adhered to the membranes were fixed with a 1:1 mixture of formaldehyde (2.5%) and glutaraldehyde (2.5%). Post-fixation was performed using osmium tetroxide: PBS (1:1). The samples were then dehydrated using a graded ethanol series (50, 70, 80, 90, 95, and 100%). After critical-point drying, the membranes were sputter-coated with platinum and observed using an SEM (S-4800 SEM, Hitachi, Tokyo, Japan) at an accelerating voltage of 10 kV.

### 3.10. Statistical Analysis

The analysis was performed by one-way ANOVA followed by Dunnett’s test, the Mann–Whitney test, or the Kruskal–Wallis test in SPSS version 23 (SPSS Inc., Chicago, IL, USA). The numbers of repetitions for each assay are given above, and results are presented as the means ± standard deviations (SD). *P* values of <0.05 or <0.01 (as indicated) were considered significant.

## 4. Conclusions

CS-g-poly (MA-co-AN) was successfully synthesized and further embedded with HA as a complex by ionotropic gelation method and the complex CS-g-poly (MA-co-AN) HA was evaluated against *C. albicans*-associated biofilm to explore its application in the medical sector. The modified CS, copolymerized by using MA and AN inhibited *C. albicans* biofilm formation. In order to support our proposed hypothesis, we found decent outcomes of present research that prepared CS-g-poly (MA-co-AN) HA complex might be an effective alternative for treating *C. albicans* biofilm-associated infections. Interestingly, CS-g-poly (MA-co-AN) HA with cationic surface charge exhibited outstanding penetration ability inside the *C. albicans* biofilm in vitro. Therefore, the cationic surface-charged polymer composite adheres to the *C. albicans*, inducing membrane permeabilization. CS-g-poly (MA-co-AN) HA polymer-treated *C. albicans* cells showed inhibition of hyphal cell formation and a reduction of adherence ability to abiotic surfaces. Furthermore, the high dose safety supports and effectiveness of CS-g-poly (MA-co-AN) HA against an established biofilm with a fluconazole-resistant strain might allow further applications of CS-g-poly (MA-co-AN) HA along with a combination of other fungicidal drugs to treat biofilm-associated chronic infections. Thus, CS-g-poly (MA-co-AN) HA complex, based on its significant applications for the treatment of *C. albicans*-associated biofilm, might be used for the drug delivery application of antifungal drugs.

## Data Availability

Not applicable.
